# Dynamic electrophysiological changes in abnormal brain cavities post-ischemic stroke

**DOI:** 10.3389/fnins.2025.1565255

**Published:** 2025-05-16

**Authors:** Ugur Kilic, Myles Mc Laughlin, Zhengdao Deng, Marjolijn Deprez, Nina Seminck, Boateng Asamoah, Bart Nuttin

**Affiliations:** ^1^Experimental Neurosurgery and Neuroanatomy, Department of Neurosciences, KU Leuven, Leuven, Belgium; ^2^Experimental ORL, Department of Neurosciences, KU Leuven, Leuven, Belgium; ^3^Center for Neuroscience, University of California, Davis, Davis, CA, United States; ^4^Department of Neurosurgery, UZ Leuven, Leuven, Belgium

**Keywords:** stroke recovery, encephalomalacia, oscillatory power, cortical lesions, local field potentials, event-related potentials

## Abstract

**Introduction:**

Stroke is a global health challenge and the leading cause of long-term disability. While survival rates have improved, effective treatments for post-stroke impairments remain lacking. A novel approach to address this unmet need involves targeting the cavities that develop after ischemic events, referred to as abnormal brain cavities (ABCs), for post-stroke neuromodulation. Despite their potential significance, ABCs have not been systematically studied, creating a gap in understanding their role in recovery and therapeutic strategies. This study represents the first investigation into the electrophysiological properties of ABC walls.

**Methods:**

To explore this, we developed an ABC model in anesthesized rats (male, *n* = 11) through controlled aspirations of the forelimb area of the motor cortex. We recorded local field potentials (LFPs), event-related potentials (ERP), and spiking activity across various conditions, including healthy, acute, and chronic phases from different anatomical locations of the ABC wall.

**Results:**

Our findings revealed significant effects of both location and condition on oscillatory power across different frequency bands. We observed significant decreases in power across different conditions (*p* < 0.0001), and this decrease varied in different locations. Similarly, our analysis showed significant effects of location and condition on ERP amplitudes, revealing a marked reduction in the acute phase (*p* = 0.001), followed by recovery in the chronic phase (*p* = 0.007). As the condition progressed to the chronic phase, these ERPs had shorter latencies (*p* < 0.0001). Notably, our results demonstrated that spiking rates remained consistent, across different conditions.

**Discussion:**

This near-normal single-unit activity suggests that the ABC wall has the potential to serve as an effective interface for neuromodulation. Additionally, the significant effects of location on our outcome measures indicates that, location-specific electrophysiologic signatures exist within the ABC wall, which could guide targeted stimulation strategies. Overall, this study underscores the need for further research into stimulation techniques targeting ABCs to facilitate recovery in stroke patients, as the ABC wall presents a promising opportunity for direct access to lesioned brain areas.

## 1 Introduction

Stroke remains a pervasive and unresolved global challenge, causing severe impairments and significantly reducing the quality of life for patients and their surroundings (Miller et al., 2010; Donkor, 2018; Benjamin et al., 2019; Tsao et al., 2023). Annually, 12 million people are diagnosed with stroke (Feigin et al., 2021). While stroke-related fatalities have decreased, the aging population is projected to lead to a 27% increase in the number of individuals living with stroke by 2050 (Vos et al., 2020; Feigin et al., 2021). Consequently, substantial resources are being directed toward stroke research and treatment. Electric brain stimulation has proven realtively successful in diseases such as tremor, epilepsy and Parkinson's disease (Salanova et al., 2015; Mahlknecht et al., 2022; Denison and Morrell, 2022). As a result, current stroke research is exploring novel neuromodulation techniques to target the nervous system (e.g., electric stimulation of the vagus nerve, the motor cortex and the spinal cord) as a therapy (Buch et al., 2008; Levy et al., 2016; Dawson et al., 2016; Abbasi et al., 2021; Kimberley et al., 2018). Despite ongoing efforts, stroke impairments remain remarkably resistant to intervention. Clinical trials show that techniques such as repetitive transcranial magnetic stimulation (rTMS) and epidural cortical stimulation are not superior to traditional rehabilitation modalities such as physiotherapy (Hsu et al., 2012; Nowak et al., 2008; Levy et al., 2016). This resistance may primarily stem from a lack of specificity in targeting intricate shifts in neural dynamics within the affected areas (Ting et al., 2021; Ganguly et al., 2022). A more comprehensive understanding of stroke-induced neurophysiological changes will benefit current neuromodulation strategies (Dawson et al., 2024).

A majority of stroke studies primarily focus on broad changes around ischemic lesions at the hemisphere level and have significantly shaped our understanding of the pathology (Leonardi et al., 2022). For example, Ramanathan et al. showed that, after power loss, the regain of low-frequency oscillations in the motor cortex (M1) correlated with recovery (Ramanathan et al., 2018). However, as cerebral infarcts progress beyond the acute phase, they often leave behind cerebrospinal fluid (CSF)—containing lesions or abnormal brain cavities (ABC). This occurs in over 90% of cerebral infarcts (Moreau et al., 2012; Loos et al., 2012). Despite their growing prevalence, our understanding of these ABCs remains limited, likely due to challenges associated with accessing their complex surfaces or walls. In this study, we define the ABC wall as the tissue immediately surrounding the post-stroke cavity. This wall is characterized by gliotic transformation and possibly the presence of preserved neurons. The development of new flexible and highly compliant brain stimulation electrodes finally enable us to investigate ABCs more thoroughly (Zhao et al., 2023a,b; Hong and Lieber, 2019; Chung et al., 2019). Therefore, it is now time to gain deeper insight into the complex post-stroke changes in the ABC wall to better leverage the capabilities of these novel electrodes.

In this study, we aimed to identify the electrophysiological signatures of the ABC wall. We first developed a rat model of an iatrogenic abnormal brain cavity (ABC) by controlled aspiration of the forelimb area of the motor cortex. We then recorded local-field potentials (LFP), event-related potentials (ERP), and single-unit activity from multiple parts of the ABC wall under different conditions (various conditions of the injury). We show that following the injury the ABC wall underwent electrophysiological changes which were specific in time (condition) and space (ABC wall location).

## 2 Materials and methods

### 2.1 Animals

All animal work was approved by the KU Leuven Ethical Committee for Animal Experimentation under Belgian legislation (Royal Decree regarding the protection of laboratory animals of 29 May 2013) and European directive (2010/63/EU) (Project number: P124/2011 and P103/2021). We used male Sprague-Dawley (SD) rats (*n* = 23, 300–350 g; Charles River Laboratories, Germany) that were housed in pairs in a 14/10-h day/night cycle (lights off at 9 PM).

### 2.2 Experimental design

**Figure 1:**
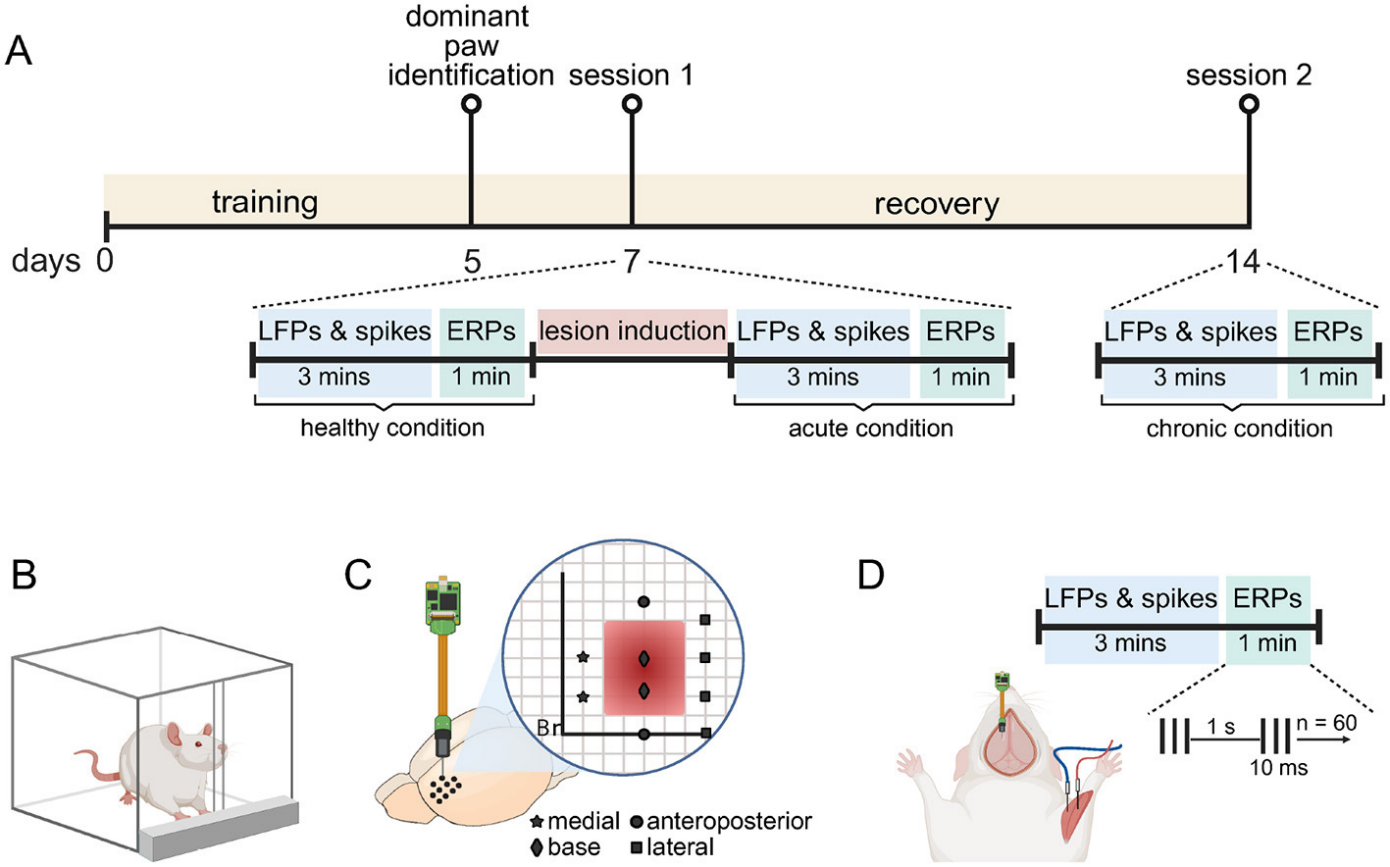
Experimental design. **(A)** Timeline of the experimental procedure. Rats were trained to perform the pellet-reaching task in the first 5 days. We used this training to identify the dominant forelimb on day 5. On day 7, they underwent recording session 1 under ketamine anesthesia. During this session, we first recorded spontaneous neural activity (LFP and spikes) over 3 min and then event-related potential (ERP) over a 1 min period in the healthy motor cortex. We then induced the abnormal brain cavity (lesion) by aspirating parts of the motor cortex. Immediately after induction, recordings were repeated for the acute condition. After a 1 week recovery period, we repeated the recordings for the chronic condition (day 14). **(B)** Dominant forepaw was determined using a single-pellet reaching task. Rats were food-restricted and trained to retrieve food pellets through a narrow slit, ensuring the engagement of a preferred forelimb. **(C)** Recordings were performed using a 32-channel silicon probe. Stereotactic coordinates for probe penetrations are indicated on a virtual grid by four different symbols: star (medial), square (lateral), circle (anteroposterior), and diamond (base). Penetrations marked by the same symbol were later grouped as an ABC wall for further analysis. **(D)** Setup for a recording. Each recording session consisted of a 3 min LFP and spike recording, followed by a 1 min ERP recording. ERPs were elicited using a train of three electrical pulses (10 ms duration at 300 Hz, pulse duration: 100 μs), delivered at 1 Hz for a total of 60 trials. The cathode and anodes were placed in the flexor and extensor muscles of the dominant forepaw via custom-made needle electrodes. This figure is Created in BioRender (Kilic, 2025). (https://BioRender.com/u19amp5).

In this work we aimed to record and track electrophysiological changes resulting from an abnormal brain cavity (ABC). We first generated a functional map of the motor cortex (M1) to guide us in creating the ABC lesion (see Supplementary Figure S1 for details of this experiment). In this paragraph, we sketch the experimental design; further details are given in subsequent method sections. We first trained rats, over a period of 5 days, on a single-pellet reaching task to determine their dominant forelimb over 5 days (see Figures 1A, B) (Whishaw et al., 1992). Two days later, rats were anesthetized and we conducted electrophysiology session one (1), where we recorded from the healthy M1 (healthy condition) using a 32-channel silicon probe. We performed 10 probe penetrations at different M1 locations (see Figure 1C). In each location we recorded spontaneous neural activity and then event related potentials (ERPs) before proceeding to the next location. Following healthy condition recordings, we created the ABC at pre-determined coordinates (see Section 2.4. Motor cortex aspiration; see Figure 1C) and immediately repeated the spontaneous neural and ERP recordings in this acute condition in the same 10 penetration locations. After acute recordings we concluded session one and allowed animals to recover (see Figure 1A for timeline details). In session two (2), which was conducted seven days after session one, we recorded for the chronic condition. To do this we used the same 32-channel silicon probe to perform the same spontaneous and ERP activity recordings at the same penetration locations. At the end of session two rats were perfused for the purpose of histology (see Section 2.7 Perfusion and histology; Supplementary Figure S2).

### 2.3 Surgery

We anesthetized rats with a ketamine/xylazine mixture (respectively, 100 mg/kg; Nimatek, Dechra, England, and 10 mg/kg; Domitor, Orion Pharma, Finland). We monitored anesthesia levels using the foot pich reflex and breathing rate and maintained anesthesia through regular ketamine/xylazine injections. We placed rats in a stereotactic frame (Stereotaxic-U frame, WPI) and exposed the skull to visualize the skull sutures. For the purpose of recording and creating the ABC we then created a craniotomy (anterior: 4 mm, posterior: −1 mm, lateral: 4.5 mm). To provide perioperative pain care, rats received an injection of lidocaine (1 ml; Xylocaine 2%, Aspen Pharma Trading Limited, Ireland) at incision sites. Furthermore, we administered a dose of meloxicam (2 mg/kg; Metacam, Boehringer Ingelheim, Germany) daily for 3 days post surgery. In non-recovery experiments, rats were either euthanized with an i.p. overdose of sodium pentobarbital (250 mg/kg; Dolethal, Vetoquinol, England) or perfused with intracardial formaldehyde (see Section 2.7. Perfusion and Histology).

### 2.4 Motor cortex aspiration

On the basis of the functional map (see Supplementary Figure S1), we created an iatrogenic ABC in the motor cortex contralateral to the dominant limb. To do this, we aspirated an area spanning 1.5–3 mm ML, 0.5–3 mm AP (relative to Bregma), and 1.5 mm depth (measured from the pial surface). For the aspiration, we used a 1 mm tipped blunt Fergusson suction cannula (B.Braun Melsungen AG) secured to a stereotactic microdrive and attached to a vacuum pump (HYVAC2, Central Instruments Corporation, USA).

### 2.5 Electrophysiology setup

Electrophysiological recordings were performed using a single-shank silicon probe with 32 channels spanning 1,550 μm (Model: E32+R-50-S1-L10 NT, Atlas Neuro, Leuven, Belgium). All electrodes on the probe were organized in one column. The probe was slowly lowered at a controlled rate of approximately 2 μm/s to a depth of 1,800 from the pial surface. We inserted a surgical bone screw through the skull to touch the CSF; this served as the recording reference. The entire setup was enclosed within a Faraday cage to reduce electric noise in the recording. Neural signals were amplified ( × 192), digitized (16 bit and 30 kHz sampling rate) and band-pass filtered (0.1–5 kHz) using a 32-channel low-noise amplifier (RHD recording headstage; Intan Technologies LLC, Los Angeles, CA) and the Open Ephys Acquisition Board (http://www.open-ephys.org/). The recordings were visualized using the Open Ephys GUI (https://open-ephys.github.io/gui-docs/) and stored on a hard drive for offline analysis.

### 2.6 Recording and stimulation

After inserting the recording probe into a target location (see Figure 1C), we first recorded spontaneous neural activity for 3 min. We used this 3 min recording for local field potential (LFP) and spiking analysis. We then proceeded to record event-related potentials (ERP). To induce ERP's we inserted custom-made needle electrodes into the flexor and extensor of the contralateral forelimb (see Figure 1D). We stimulated with biphasic pulses (1 mA, pulse width: 100 μs for each phase) at 300 Hz for a duration of 10 ms. We repeated this stimulation every second for 60 s. This resulted in a train of three pulses which caused a limb movement and a subsequent ERP every second, thereby giving a total of 60 ERP's per recording location. To deliver peripheral electric stimulation, the cathode and anode terminals of a current source (Model 2200; AM Systems, Sequim, WA) were connected to the needle electrodes in the flexor and extensor, respectively. The current source was driven by an analog signal generated by a data acquisition card (NI USB-6216, National Instruments, Austin, TX), which operated at a sampling rate of 30 kHz. The data acquisition card was controlled via a custom-written, Matlab 2021b (MathWorks, Natick, MA) based software. During recordings, anesthesia depth was kept constant by continuously monitoring and assessing respiratory rate and the foot-pinch reflex.

### 2.7 Perfusion and histology

At the end of the second recording session, rats were intracardially perfused with saline and 4% formaldehyde. Brains were then immersed in 4% formaldehyde (Sigma Aldrich, Merck, Germany) for 24 h. After this, they were embedded in paraffin, and coronal slices (5 μm thick) were cut and stained using the hematoxylin and eosin protocol (see Supplementary Figure S2).

### 2.8 Data processing and analysis

#### 2.8.1 Local field potentials

For LFP analysis we bandpass filtered (0.5–250 Hz) data from the 3 min spontaneous recordings (see Section 2.6 Recording and stimulation) using a zero-phase, second-order Butterworth filter. To compute spectral power, we applied a short-time Fourier transform using a Hann window of five seconds with 50% overlap. Within predefined frequency bands: delta (0.5–4 Hz), theta (4–7 Hz), alpha (7–13 Hz), beta (13–30 Hz), low-gamma (30–60 Hz), and high-gamma (60–90 Hz) the acquired frequency components were averaged to get band power (Berens et al., 2008; Colgin, 2016; Nayak and Anilkumar, 2023). For each penetration location (see Figure 1C) we repeated this procedure for all 32 recording channels. We then averaged the band power (separately for each band) across all 32 channels to get the power for that specific penetration location.

#### 2.8.2 Event-related potentials

**Figure 2:**
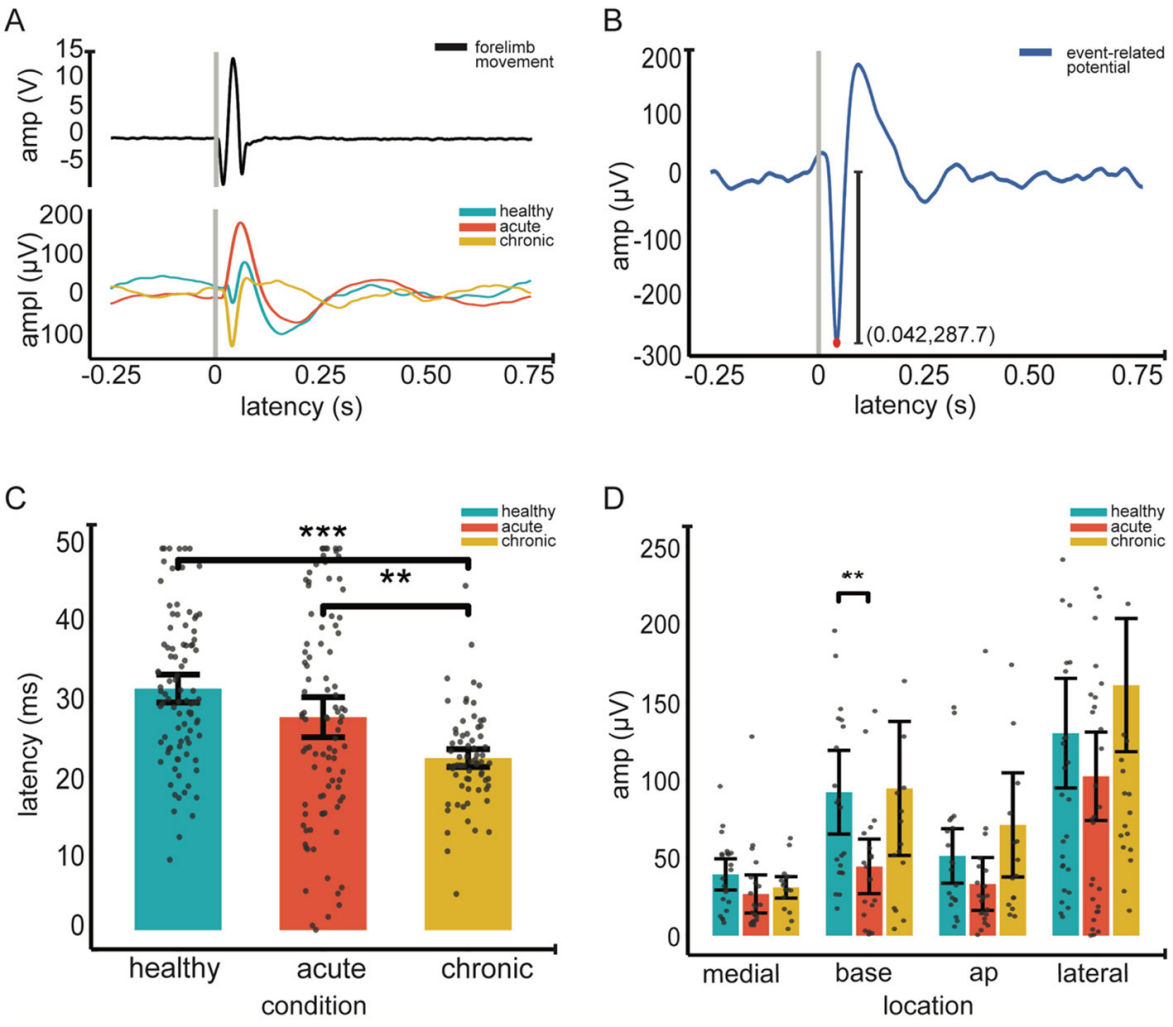
Event-related potentials (ERP) across conditions. **(A)**. Top Individual example of an average forelimb movement in response to peripheral electrical stimulation, recorded via an accelerometer. Gray vertical line marks the stimulation onset (time 0); epoch duration is 1 second. Bottom individual grand average ERP waveforms for healthy (blue), acute (orange) and chronic (yellow) conditions (across 60 epochs and 32 channels). **(B)**. Extraction of ERP amplitude and latency An example grand average ERP showing the extraction of the latency (red dot) and the amplitude (vertical line) (amplitude, 287.8 μV and latency, 42 ms) within the 0–50 ms post stimulation window. **(C)** Mean ERP latency across conditions. **(D)** Mean ERP amplitude across recording locations and conditions. In **(C, D)**, gray dots represent individual data points; error bars indicate 95% confidence intervals, Significance was assessed using linear-mixed effect models followed by *post hoc* pairwise comparisons with Tukey correction (12 total). Asterisks indicate significance; **p < 0.01, *** < 0.001.

For ERP analysis we used a zero-phase, second-order Butterworth filter to bandpass filter (1–300 Hz) the recorded ERP data (see Section 2.6 Recording and stimulation). For each of the 32 recording channels we averaged all 60 epochs to get one channel average. We ensured this channel average centered around zero by subtracting the channel average from each of the 60 ERP's. We then averaged all 32 channel averages to get a grand average ERP which represented that recording location. To determine peak-to-peak amplitude and latency, we first identified the trough within the first 50 ms following stimulation onset. The subsequent ERP peak was then identified within the 50–80 ms window from stimulation onset. The zero to trough amplitude was then calculated as the absolute difference between these two values. We defined latency as the time at which the ERP trough occurred (see Figure 2B).

#### 2.8.3 Action potentials

For spike sorting we first bandpass filtered (300–3,000 Hz) the 3 min spontaneous recording (see Section 2.6 Recording and stimulation) using a zero-phase second-order Butterworth filter which we then fed to Spyking Circus (https://spyking-circus.readthedocs.io/en/latest/#). Via a series of automatic steps Spyking Circus detected action potentials and clustered them on the basis of spike features, spike timing as well as spike spatial position (Yger et al., 2018). Following this automatic sorting, we manually curated clusters to get well isolated units. To do this we checked whether cluster waveforms resembled spike waveforms and ensured the cluster had a clear refractory period. We further ensured spike shape had little variability (Hill et al., 2011). We took all clusters that survived this manual curation as putative single units and did not further split them on the basis of waveform characteristics (e.g., fast- and regular-spiking neurons). For each putative single unit, we extracted spike times and then computed its firing rate by dividing the number of spikes over the length of the recording. For statistical analysis all single units were grouped per recording location (one of the ten recording positions).

### 2.9 Statistics

Our data contains repeated measurements for each animal and recording site separately. This design creates dependence and correlation between some observations. To address this we applied linear and generalized linear mixed models (Yu et al., 2022). We fitted models for LFP power spectral density (PSD), ERP amplitude and latency, as well as spike rate. Before fitting the models, we visually inspected data distributions, performed goodness-of-fit tests, and examined quantile-quantile plots for residuals to ensure model assumptions were met. When necessary, data transformations were applied to improve normality and homoscedasticity.

For PSD, the data were log-transformed for better goodness-of-fit, and we fitted an LMM with condition (healthy, acute, chronic) and location (medial, lateral, base, anteroposterior) as fixed effects and random intercepts for individual animals [Power ~ Condition × Location + (1 | Rat)]. A separate model was fitted for each frequency band. For ERP data, separate LMMs were fitted for amplitude [Amplitude ~ Condition × Location + (1 | Rat)] and latency [Latency ~ Condition × Location + (1 | Rat)] with amplitude values log-transformed to improve normality, while latency remained untransformed. Because spiking data is discrete we used a GLMM. We chose the gamma family distribution based on visual inspection and goodness-of-fit tests. We then used the same fixed and random effects structure as for the LFP and ERP analyses [Spike Rate ~ Condition × Location + (1 | Rat)].

Chi-square tests were performed on all model outputs to assess the significance of fixed effects. *Post-hoc* comparisons were conducted for three conditions (healthy, acute, chronic) and four locations (medial, lateral, base, anteroposterior), resulting in 12 comparisons. Tukey's honestly significant difference (HSD) test was applied to correct for multiple comparisons across these 12 conditions. All statistical analyses were performed in RStudio [R version: 4.2.3 (2023-03-15); RStudio version: 2023.03.0 + 386, Posit Software, PBC].

## 3 Results

In this study, we aimed to understand location-specific electrophysiological adaptations within the ABC wall. Using an anesthetized rat model, we induced an iatrogenic ABC and compared neural dynamics across three conditions: pre-lesion (healthy), immediate post-lesion (acute), and late post-lesion (chronic).

### 3.1 Band power significantly decreases in acute and chronic ABC walls

**Figure 3:**
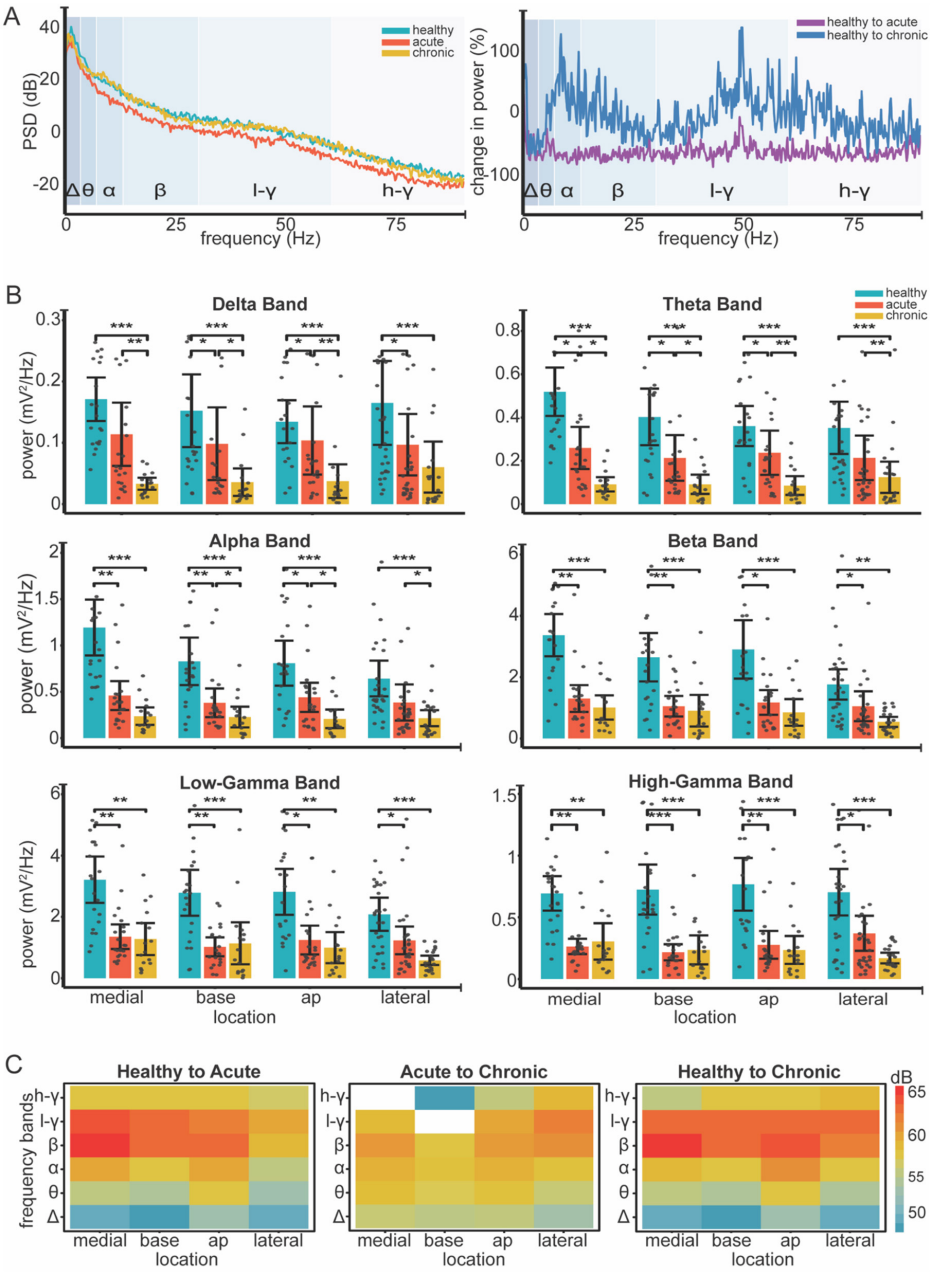
Changes in LFP band power across different locations and conditions. **(A)** Left Individual example of power spectral density (PSD) across healthy (blue), acute (orange) and chronic (yellow) conditions. Log transformation was used to visualize all frequency bands on a unified scale. Right Normalized change in power (%) relative to healthy condition calculated as (condition-healthy) / healthy * 100. **(B)** Bar plots show mean power for all frequency band across recording locations and conditions (healthy, acute, chronic). Color coding is consistent across plots, as the legend indicates (top right-panel **B)**. Gray dots represent individual data points; error bars indicate 95% confidence intervals. Significance was assessed using linear mixed-effect models, followed by *post-hoc* pairwise comparisons with Tukey correction (12 total). Asterisks indicate significance levels: * < 0.05, ** < 0.01, *** < 0.001. **(C)** Heatmaps show absolute differences [in decibels (dB)] in power between conditions: healthy to acute, acute to chronic, and healthy to chronic. Red shades represent greater power decreases, while blue shades indicate smaller decreases. White areas indicate power increases from acute to chronic. Log transformation was used to enable consistent color representation across conditions.

We used LMM to measure the effect of condition and location on the band power extracted from the 3 min spontaneous recordings. Firstly, our analysis revealed a significant effect of condition on the power of individual frequency bands (see Table 1 for Statistics). Pairwise comparisons showed a significant decrease in band power across all frequencies from healthy to acute conditions (*see* Table 2 for Statistics). With the exception of the high gamma band (*p* = 0.06), all bands showed a further decrease in power from the acute to chronic condition (see Table 2 for Statistics) (see Figure 3A).

**Table 1:** Main effects on band power differences.

**Frequency bands**	**χ^2^ (Df)**	***p*-value**	**χ^2^ (Df)**	***p*-values**
Delta	***χ***^2^ (2) = 101.14	< 0.0001	***χ***^2^ (3) = 3.24	NS (0.35)
Theta	***χ***^2^ (2) = 124.77	< 0.0001	***χ***^2^ (3) = 6.11	NS (0.10)
Alpha	***χ***^2^ (2) = 117.19	< 0.0001	***χ***^2^ (3) = 8.01	0.04
Beta	***χ***^2^ (2) = 81.36	< 0.0001	***χ***^2^ (3) = 10.47	0.01
Low gamma	***χ***^2^ (2) = 78.1	< 0.0001	***χ***^2^ (3) = 7.29	NS (0.06)
High gamma	***χ***^2^ (2) = 95.5	< 0.0001	***χ***^2^ (3) = 1.59	NS (0.66)

Chi-squared (χ^2^) test statistics, degrees of freedom (df), and p-values are reported for fixed effects of condition and location in linear mixed-effects models (LMER), applied separately to local field potential (LFP) power in each frequency band. NS, not significant.

**Table 2:** Pairwise comparisons of LFP band power by conditions.

**Frequency bands**	**Healthy vs. acute**			**Acute vs. chronic**			**Healthy vs. chronic**		
	**Est**.	**SE**	***p*** **-value**	**Est**.	**SE**	***p*** **-value**	**Est**.	**SE**	***p*** **-value**
Delta	0.28	0.16	< 0.0001	0.96	0.17	< 0.0001	1.79	0.17	< 0.0001
Theta	0.88	0.14	< 0.0001	0.94	0.16	< 0.0001	1.82	0.16	< 0.0001
Alpha	0.92	0.14	< 0.0001	0.76	0.15	< 0.0001	1.69	0.14	< 0.0001
Beta	0.99	0.15	< 0.0001	0.48	0.17	0.01	1.48	0.17	< 0.0001
Low gamma	0.92	0.14	< 0.0001	0.42	0.16	0.02	1.34	0.16	< 0.0001
High gamma	1.02	0.13	< 0.0001	0.35	0.15	NS (0.06)	1.37	0.15	< 0.0001

Estimated marginal means (Est.), standard errors (SE), and p-values are reported for *post hoc* pairwise comparisons between conditions (Healthy vs. Acute, Acute vs. Chronic, and Healthy vs. Chronic) following linear mixed-effects models. p-values were adjusted using Tukey's method for 12 multiple comparisons. NS: not significant.

Our analysis revealed significant effects of condition on band power as well as distinct patterns of power alterations at different locations during various phases of the ABC wall (see Figure 3B). The power decrease from the healthy to acute condition did not show location-specific patterns, whereas the transition from acute to chronic displayed variations that depended on the recording site.Particularly in the lateral wall, band power was preserved in the chronic phase (see Table 2 for statistics), unlike other walls where power continued to decline in the low-frequency bands (delta, theta, and alpha) (see Table 3 for Statistics). We further measured the main effect of location on power, identifying significant differences in the alpha and beta frequency bands (*p* = 0.04 and *p* = 0.01, respectively) (see Figure 3C). These results suggest that band power, is differentially modulated across distinct regions of the ABC wall and varies with both the phase and the specific location of the recordings.

**Table 3:** *Post hoc* comparisons of band power by condition and location.

**Location**	**Frequency bands**	**Healthy vs. acute**			**Acute vs. chronic**			**Healthy vs. chronic**		
		**Est**.	**SE**	***p*** **-value**	**Est**.	**SE**	***p*** **-values**	**Est**.	**SE**	***p*** **-value**
Medial wall	Delta	0.74	0.32	**0.05**	1.05	0.35	**< 0.01**	1.78	0.35	**< 0.0001**
	Theta	0.92	0.29	**0.01**	0.99	0.32	**0.01**	1.91	0.32	**< 0.0001**
	Alpha	1.07	0.28	**< 0.001**	0.71	0.31	**NS (0.05)**	1.79	0.31	**< 0.0001**
	Beta	1.18	0.3	**< 0.001**	0.32	0.33	**NS (0.61)**	1.5	0.33	**0.0001**
	L-gamma	0.95	0.28	**< 0.01**	0.27	0.31	**NS (0.66)**	1.22	0.32	**< 0.001**
	H-gamma	0.98	0.27	**< 0.01**	0.16	0.3	**NS (0.84)**	1.14	0.3	**< 0.001**
Base	Delta	0.80	0.31	**0.03**	0.98	0.35	**0.01**	1.78	0.35	**< 0.0001**
	Theta	0.89	0.29	**0.01**	0.94	0.32	**0.01**	1.84	0.32	**< 0.0001**
	Alpha	0.92	0.28	**< 0.01**	0.76	0.31	**0.04**	1.68	0.31	**< 0.0001**
	Beta	1.1	0.3	**0.001**	0.45	0.33	**NS (0.36)**	1.56	0.33	**< 0.0001**
	L-gamma	1.13	0.28	**< 0.001**	0.29	0.31	**NS (0.61)**	1.43	0.31	**< 0.0001**
	H-gamma	1.29	0.27	**< 0.0001**	0.2	0.3	**NS (0.76)**	1.5	0.30	**< 0.0001**
Anteroposterior wall	Delta	0.78	0.32	**0.04**	1.11	0.35	**0.01**	1.89	0.35	**< 0.0001**
	Theta	0.82	0.3	**0.01**	1.13	0.32	**< 0.01**	1.95	0.32	**< 0.0001**
	Alpha	0.87	0.29	**0.01**	0.94	0.31	**0.01**	1.81	0.31	**< 0.0001**
	Beta	0.92	0.31	**0.01**	0.55	0.33	**NS (0.23)**	1.48	0.34	**0.0001**
	L-gamma	0.87	0.29	**0.01**	0.41	0.31	**NS (0.39)**	1.29	0.32	**< 0.001**
	H-gamma	1.07	0.28	**< 0.001**	0.32	0.3	**NS (0.53)**	1.4	0.3	**< 0.0001**
Lateral wall	Delta	0.98	0.31	**0.01**	0.72	0.35	**NS (0.1)**	1.7	0.34	**< 0.0001**
	Theta	0.88	0.29	**0.01**	0.69	0.32	**NS (0.08)**	1.57	0.32	**< 0.0001**
	Alpha	0.82	0.28	**0.01**	0.64	0.31	**NS (0.1)**	0.46	0.31	**< 0.0001**
	Beta	0.76	0.3	**0.04**	0.61	0.33	**NS (0.17)**	1.37	0.33	**< 0.001**
	L-gamma	0.72	0.28	**0.03**	0.7	0.3	**NS (0.06)**	1.42	0.31	**0.0001**
	H-gamma	0.75	0.27	**0.01**	0.7	0.3	**NS (0.05)**	1.45	0.3	**< 0.0001**

Estimated marginal means (Est.), standard errors (SE), and p-values are reported for pairwise comparisons between conditions (Healthy vs. Acute, Acute vs. Chronic, and Healthy vs. Chronic) across different cortical wall regions (medial, base, anteroposterior, and lateral). p-values were adjusted using Tukey's method for multiple comparisons (12 total). NS, not significant.

### 3.2 ERP amplitude and latency vary across ABC phases

We investigated changes in the amplitude and latency of ERPs using LMMs. First, we measured the main effect of location and condition on ERP amplitudes. Our results indicate that the condition is a significant predictor of the amplitude of ERP's in the ABC wall (*p* < 0.0001). Pairwise comparisons revealed significant ERP amplitude differences between the healthy and acute conditions (*p* = 0.001) as well as the acute and chronic conditions (*p* = 0.007) (see Figure 2). Notably, ERP amplitudes in healthy and chronic ABC walls did not differ (*p* = 0.75), suggesting a rebound of ERP amplitude in the chronic phase. In addition to condition, location emerged as a significant factor affecting ERP amplitudes (*p* < 0.0001). *Post-hoc* analysis revealed that ERP amplitudes in the lateral wall differed significantly from those in other regions (lateral vs. medial wall *p* < 0.001; lateral vs. anteroposterior *p* < 0.001; lateral vs. base *p* < 0.01). (see Table 4 for Statistics). However, no significant differences were found between locations along the same sagittal plane (see Table 4 for Statistics). These findings suggest that both condition and location play crucial roles in shaping ERP responses, with the lateral wall of the ABC showing distinct amplitude patterns compared to other regions.

**Table 4:** *Post Hoc* comparisons of ERP amplitudes across locations.

**Contrast (Location vs. location)**	**Est**.	**SE**	***p*-value**
Antero-posterior vs. base	−0.22	0.2	NS (*p =* 0.68)
Antero-posterior vs. lateral	−0.91	0.2	*p < * 0.001
Antero-posterior vs. medial	0.39	0.2	NS (*p =* 0.22)
Base vs. lateral	−0.68	0.2	*p < * 0.01
Base vs. medial	0.62	0.2	*p < * 0.05
Lateral vs. medial	1.3	0.2	*p < * 0.0001

Estimated marginal means (Est.), standard errors (SE), and p-values are reported for pairwise comparisons of ERP amplitudes across locations. Values represent condition-adjusted differences derived from an LMM with condition and location as a fixed effect. *p*-values were corrected using Tukey's method for 12 comparisons.

Second, we assessed the effects of condition and location on the average latency of ERPs. Our LMM analysis identified that condition was a significant predictor of latency variations (*p* < 0.0001). Pairwise comparisons revealed that ERP latencies were significantly shorter (see Figure 2C); this change only occurred during the transition to the chronic condition [healthy to chronic conditions (*p* = 0.0001) and acute to chronic conditions (*p* = 0.02)]. In contrast, no significant latency changes were observed from the healthy to the acute phase (*p* = 0.27), indicating that latency decrease was a late onset phenomenon. Similarly, location significantly influenced latency (*p* = 0.01) as the main effect. However, *post-hoc* comparisons showed no significant differences between specific locations.

### 3.3 Single-unit firing is preserved in the ABC wall

**Figure 4:**
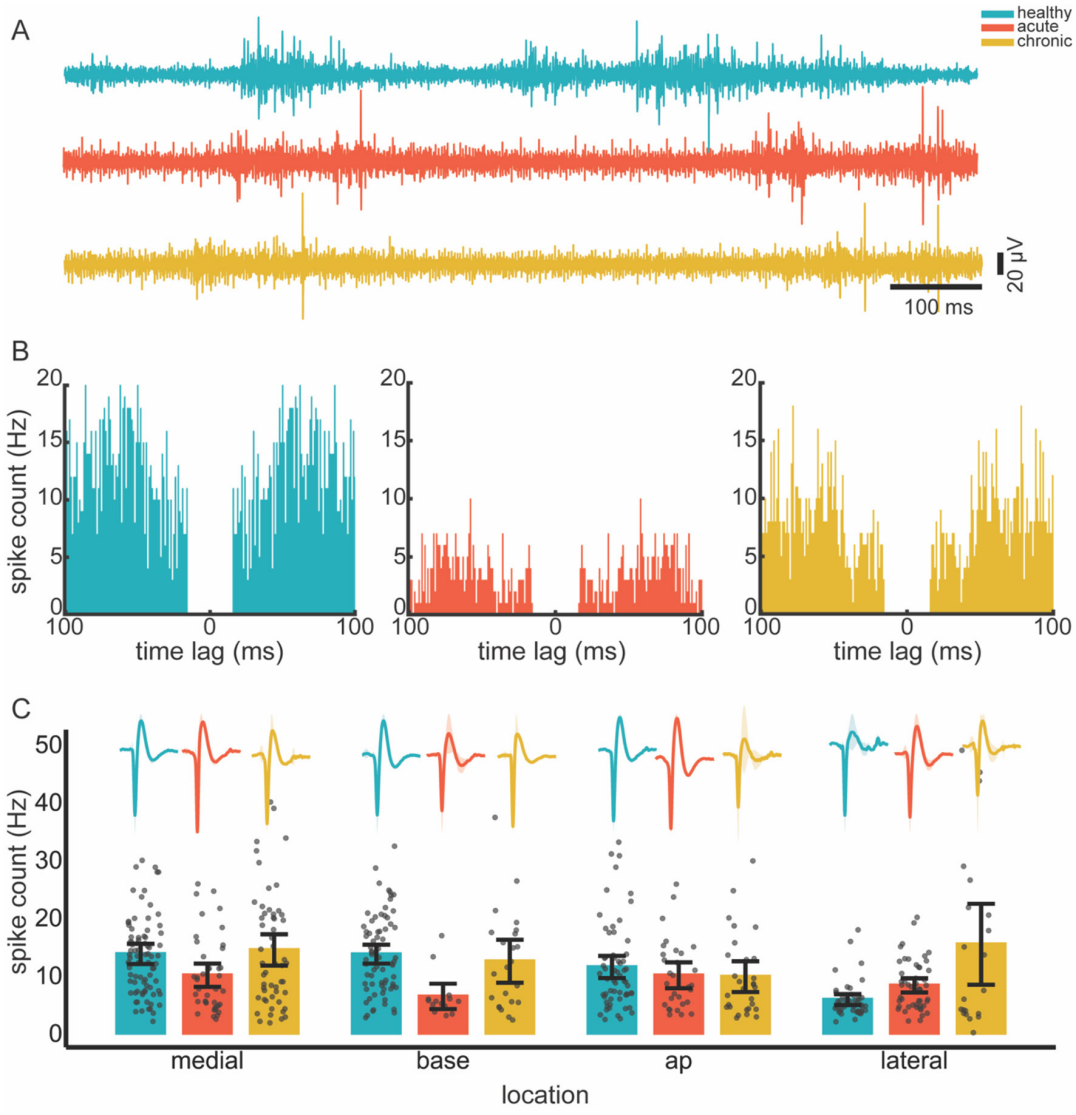
Spiking activity across conditions. **(A)** Individual example of filtered (300–3,000 Hz) neural recordings from healthy, acute and chronic conditions. **(B)** Autocorrelograms of randomly selected single units in different conditions, illustrating a clear refractory period. **(C)** Mean spike rate across recording locations (ABC wall) and conditions. Spike rate data were anlayzed using a generalized linear mixed-effect model followed by *post hoc* pairwise comparisons. Multiple comparisons were corrected by Tukey −12 times. Average spike waveforms for each condition and location are shown as inserts above the bar plots to illustrate typical unit shape. Shading around the waveforms are standard deviation. Gray dots represent individual data points; error bars show 95% confidence intervals. Color code is consistent across panels, as shown top-right.

We investigated the changes in the firing rates of the remaining units in the cavity wall. After spike sorting, we obtained 258 single units from 10 penetrations in 11 rats in healthy conditions, whereas we obtained 135 and 127 single units in the acute and chronic conditions, respectively (see Figures 4A, B). The GLMM revealed that the condition had a significant effect on the spike rate (*p* < 0.001). Location alone, however, did not significantly affect spike rates (*p* = 0.1); however, the interaction between condition and location did (*p* = 0.003). This suggests that some locations may exhibit condition-dependent changes in firing rate that are not evident when considering location as an isolated factor. Notably, however, pairwise comparisons in the *post-hoc* analysis did not detect significant differences in spike rates during the transitions from healthy to acute or acute to chronic, thereby implying spike rate changes may have been subtle (see Figure 4C).

## 4 Discussion

As the survival rate of stroke increases, more people live with abnormal brain cavities (ABCs). Until recently, accessing and studying these ABCs was challenging due to limitations in electrode flexibility and material compatibility (Zhao et al., 2023a,b; Hong and Lieber, 2019; Chung et al., 2019). In this study, we conduct the first exploration of the electrophysiological signatures of the ABC wall, thereby paving the way for advancements in electrophysiologically informed ABC wall neuromodulation in the future. We used an anesthetized rat model to investigate changes in local field potentials (LFPs), event-related potentials (ERPs), and spiking activity which enabled us to identify location-specific changes in the ABC wall across conditions.

Three outcomes of our spectral analysis are of note. Firstly, in the acute condition power decreased across the line for all frequency bands and wall locations. Since power reflects the synchronous activity of large neural populations (Gallego-Carracedo et al., 2022; Lindén et al., 2011), this power loss may indicate a dissociation within the motor cortex due to the presence of the ABC (Herreras, 2016). This power reduction was also shown in earlier stroke studies and was correlated with behavioral recovery in rodents (Rabiller et al., 2015; Ramanathan et al., 2018). This similarity of results implies that changes in neural processes can be identified without active behavior. This could be valuable for human application as ABC neurophysiology under anesthesia easily translates to stroke electrophysiology in awake experiments. Secondly, as the injury progressed from the acute to the chronic condition power in the lower bands (delta, theta, alpha) further decreased while in the higher bands (beta, high and low gamma) they stabilized. It is known that, field potentials in higher bands tend to have larger contributions of spiking activity (Buzsáki et al., 2012). As we showed in the results (see Figure 4C and Section 3.3 Single-unit firing is preserved in the ABC wall) and will discuss later on, there was a rebound of spiking activity after a decrease in the acute condition. This implies that where power in lower bands decreased over time, the contribution of spiking activity in higher power bands may have stabilized the power in these bands. Lastly, while power changes in the frequency bands (alpha and beta) were location specific this was not the case for bands below alpha where changes were similar across locations. Lower frequencies can generally carry larger distances than higher frequencies (Herreras, 2016). As a result higher frequency changes in a specific wall are more likely to remain localized whereas lower band changes can more easily become mixed over distances. Notably, alpha and beta band activities are bound to the state of the motor system. During motor dormancy both bands are dominant with beta showing transient bursts. However, upon movement initiation both are suppressed (Fransen et al., 2016; Rule et al., 2018; Feingold et al., 2015). These frequency bands may thus have been particularly vulnerable to the lesion. The location-specific effects may thus reflect the functional specialization of the bands in M1, where distinct regions contribute differently to oscillatory dynamics associated with motor function.

We further investigated the characteristics (amplitude and latency) of generated ERPs to assess ABC-related changes in motor cortex communication with the somatosensory cortex (Hatsopoulos and Suminski, 2011; Kunori and Takashima, 2016; Hishinuma et al., 2019). Compared to the healthy condition ERP amplitudes showed a marked reduction in the acute condition. From the acute to the chronic condition, the amplitudes rebounded, thereby effectively annulling the reduction in the acute condition. Since neurons are directly responsible for ERP generation (Roy et al., 2011), this acute reduction may be the direct result of neuron loss, where fewer neurons contribute to the ERP. Alternatively, connections with ERP-generating regions (e.g., corticocortical or thalamic projections) may have been disrupted (Kunori and Takashima, 2016; An et al., 2014; Fukui et al., 2020). The recovery of the ERP amplitude does imply neuron loss is the more likely explanation. From this perspective, preserved neurons—which were rendered unresponsive (e.g., due to swelling)—recovered and contributed to ERP amplitude rebound. Nevertheless, disrupted projections cannot be ruled out. As we will further argue later, hyperactivity is a common feature of brain damage; this hyperactivity has the potential to mask the effects of connection losses. Notably, ERP latencies were significantly shorter in the chronic condition. A simple recovery of unresponsive neurons alone could not explain this change in ERP latency. However, recovery combined with hyperactivity could explain how an uninhibited cortex would respond much faster than otherwise.

Similarly, spiking activity followed the ERP trend. In acute conditions, the spiking rate was reduced. However, this activity rebounded in the chronic condition. This rebound could be attributed to single units recovering in the seven days before the chronic condition recordings. This trend—which mirrored the ERP—was only obvious in the main analysis and did not survive *post-hoc* testing. This implies that the effects were more subtle at the single unit level. Importantly, our model necessitated that we removed electrodes in between conditions. As such, we were unable to track single units across conditions; rather, we pooled neural activity together within conditions and locations. Secondly, for our model, we removed the upper cortical layers. Lower cortical layers have higher firing rates (Rostami et al., 2024; Dura-Bernal et al., 2023; Quiquempoix et al., 2018); this, combined with the pooling, means the rebound could be a result of biased sampling where high rate units were overrepresented in the chronic condition as compared to the healthy. However, this biased sampling would also have occurred in the acute condition. Therefore, a plausible explanation of the rebound in spiking as well as in ERP activity is that preserved neurons recovered over the seven-day period between the first and second recording sessions.

Hyperexcitability tends to occur as a response to brain damage. For example, in stroke as well as traumatic brain injury, the balance between excitation and inhibition shifts more toward excitation (Luhmann et al., 1995; Neumann-Haefelin et al., 1995; Qü et al., 1998; Carron et al., 2016; Kim et al., 2014). This implies that excitatory neurons become more dominant in the cortex. Inhibitory neurons are innately more sensitive to deprivation of nutrients and oxygen (Zhang et al., 2008; Wang, 2003; Povysheva et al., 2019). Additionally, their influence on the network could be reduced as a result of a general loss of connections. In this first-ever exploration of the ABC wall, we did not separate single units on the basis of neural identity (e.g., fast- or regular spiking). As the technology to modulate these ABCs further develops, future studies can focus on the excitation-inhibition (E/I) balance question to leverage new insights for more effective neuromodulation.

In the current study, we used an anesthetized rodent model to focus on the walls of the ABC as a potential target for neuromodulation. To this end, we created a model (by aspiration) and tracked changes over a one-week period. This allowed us to create lesions in a consistent and replicable fashion and to record from comparable locations across conditions and subjects. Ischemic strokes cause widespread brain-blood-barrier damage with reperfusion, further exacerbating vascular damage through oxidative stress and inflammation. The vascular damage prompts long-term vascular remodeling over a period of months, which may support recovery (Jickling et al., 2014). This remodeling may be locally disrupted, maladaptive, and can lead to seizures (Dash et al., 2025; Goodman et al., 2023). Furthermore, ischemic stroke triggers robust inflammatory responses leading to secondary neurodegeneration, which extends beyond the lesion site (Jayaraj et al., 2019). Our aspiration model also caused local vascular damage and likely inflammatory responses, however, we did not assess these particular adaptations and note that there are very likely differences in damage caused, vascular remodeling and immune responses compared to an ischemic stroke.

The ketamine anesthetized cortex shows differences from the awake cortex. For example, Schroeder et al. showed that somatosensory integration is inhibited in primates (Schroeder et al., 2016). Further, Ordek et al. analyzed ECOG recordings and showed an increased correlation between neighboring electrodes in rats (Ordek et al., 2013). In the current study, as we recorded under ketamine anesthesia, we may expect differences with awake neural activity. In this study, all recordings were conducted under the same anesthesia. As such, the results between conditions can be compared with one another. Additionally, Dimitriadis et al. found that field potential propagation patterns were unaffected, and Schroeder et al. similarly observed conserved evoked potentials in the somatosensory cortex (Dimitriadis et al., 2014a,b; Schroeder et al., 2016). Our results also matched with some awake ischemic stroke studies. Ramanathan et al. reported that power decreased following stroke and showed rebound, which correlated with behavior (Ramanathan et al., 2018). This implies that, despite differences due to the anesthesia, our model remains valid in the stroke context.

## Data Availability

The raw data supporting the conclusions of this article will be made available by the authors, without undue reservation.

## References

[B1] Abbasi A. Danielsen N. P. Leung J. Muhammad A. K. M. G. Patel S. Gulati T. (2021). Epidural cerebellar stimulation drives widespread neural synchrony in the intact and stroke perilesional cortex. J. Neuroeng. Rehabil. 18:89. 10.1186/s12984-021-00881-9 34039346 PMC8157634

[B2] An S. Kilb W. Luhmann H. J. (2014). Sensory-evoked and spontaneous gamma and spindle bursts in neonatal rat motor cortex. J. Neurosci. 34, 10870–10883. 10.1523/JNEUROSCI.4539-13.2014 25122889 PMC6705262

[B3] Benjamin E. J. Muntner P. Alonso A. Bittencourt M. S. Callaway C. W. Carson A. P.. (2019). Heart Disease and Stroke Statistics-−2019 update: a report from the American Heart Association. Circulation 139, e56–e528. 10.1161/CIR.0000000000000659 30700139

[B4] Berens P. Keliris G. A. Ecker A. S. Logothetis N. K. Tolias A. S. (2008). Feature selectivity of the gamma-band of the local field potential in primate primary visual cortex. Front. Neurosci. 2, 199–207. 10.3389/neuro.01.037.2008 19225593 PMC2622750

[B5] Buch E. Weber C. Cohen L. G. Braun C. Dimyan M. A. Ard T.. (2008). Think to Move: a Neuromagnetic Brain-Computer Interface (BCI) System for Chronic Stroke. Stroke 39, 910–917. 10.1161/STROKEAHA.107.505313 18258825 PMC5494966

[B6] Buzsáki G. Anastassiou C. A. Koch C. (2012). The origin of extracellular fields and currents—EEG, ECoG, LFP and spikes. Nat. Rev. Neurosci. 13, 407–420. 10.1038/nrn3241 22595786 PMC4907333

[B7] Carron S. F. Alwis D. S. Rajan R. (2016). Traumatic brain injury and neuronal functionality changes in sensory cortex. Front. Syst. Neurosci. 10, 47. 10.3389/fnsys.2016.00047 27313514 PMC4889613

[B8] Chung J. E. Joo H. R. Fan J. L. Liu D. F. Barnett A. H. Chen S.. (2019). High-density, long-lasting, and multi-region electrophysiological recordings using polymer electrode arrays. Neuron 101, 21–31.e5. 10.1016/j.neuron.2018.11.002 30502044 PMC6326834

[B9] Colgin L. L. (2016). Rhythms of the hippocampal network. Nat. Rev. Neurosci. 17, 239–249. 10.1038/nrn.2016.21 26961163 PMC4890574

[B10] Dash U. C. Bhol N. K. Swain S. K. Samal R. R. Nayak P. K. Raina V.. (2025). Oxidative stress and inflammation in the pathogenesis of neurological disorders: mechanisms and implications. Acta Pharmaceutica Sinica B 15, 15–34. 10.1016/j.apsb.2024.10.004 40041912 PMC11873663

[B11] Dawson J. Abdul-Rahim A. H. Kimberley T. J. (2024). Neurostimulation for treatment of post-stroke impairments. Nat. Rev. Neurol. 20, 259–268. 10.1038/s41582-024-00953-z 38570705

[B12] Dawson J. Pierce D. Dixit A. Kimberley T. J. Robertson M. Tarver B.. (2016). Safety, feasibility, and efficacy of vagus nerve stimulation paired with upper-limb rehabilitation after ischemic stroke. Stroke, 47, 143–150. 10.1161/STROKEAHA.115.010477 26645257 PMC4689175

[B13] Denison T. Morrell M. J. (2022). Neuromodulation in 2035: The neurology future forecasting series. Neurology 98, 65–72. 10.1212/WNL.0000000000013061 35263267 PMC8762584

[B14] Dimitriadis G. Fransen A. M. Maris E. (2014a). Sensory and cognitive neurophysiology in rats, Part 1: Controlled tactile stimulation and micro-ECoG recordings in freely moving animals. J. Neurosci. Methods 232, 63–73. 10.1016/j.jneumeth.2014.05.001 24820913

[B15] Dimitriadis G. Fransen A. M. M. Maris E. (2014b). Sensory and cognitive neurophysiology in rats. Part 2: validation and demonstration. J. Neurosci. Methods 232, 47–57. 10.1016/j.jneumeth.2014.05.002 24814253

[B16] Donkor E. S. (2018). Stroke in the 21(st) century: a snapshot of the burden, epidemiology, and quality of life. Stroke Res. Treat. 2018, 3238165. 10.1155/2018/3238165 30598741 PMC6288566

[B17] Dura-Bernal S. Neymotin S. A. Suter B. A. Dacre J. Moreira J. V. S. Urdapilleta E.. (2023). Multiscale model of primary motor cortex circuits predicts *in vivo* cell-type-specific, behavioral state-dependent dynamics. Cell Rep. 42:112574. 10.1016/j.celrep.2023.112574 37300831 PMC10592234

[B18] Feigin V. L. Krishnamurthi R. V. Stark B. A. Johnson C. O. Roth G. A. Bisignano C.. (2021). Global, regional, and national burden of stroke and its risk factors, 1990–2013; 2019: a systematic analysis for the Global Burden of Disease Study 2019. Lancet Neurol. 20, 795–820. 10.1016/S1474-4422(21)00252-0 34487721 PMC8443449

[B19] Feingold J. Gibson D. J. DePasquale B. Graybiel A. M. (2015). Bursts of beta oscillation differentiate postperformance activity in the striatum and motor cortex of monkeys performing movement tasks. Proc. Natl. Acad. Sci. USA. 112, 13687–13692. 10.1073/pnas.1517629112 26460033 PMC4640760

[B20] Fransen A. M. Dimitriadis G. van Ede F. Maris E. (2016). Distinct α- and β-band rhythms over rat somatosensory cortex with similar properties as in humans. J. Neurophysiol. 115, 3030–3044. 10.1152/jn.00507.2015 27009160 PMC4922619

[B21] Fukui A. Osaki H. Ueta Y. Kobayashi K. Muragaki Y. Kawamata T.. (2020). Layer-specific sensory processing impairment in the primary somatosensory cortex after motor cortex infarction. Sci. Rep. 10, 3771. 10.1038/s41598-020-60662-7 32111927 PMC7048762

[B22] Gallego-Carracedo C. Perich M. G. Chowdhury R. H. Miller L. E. Gallego J. Á. (2022). Local field potentials reflect cortical population dynamics in a region-specific and frequency-dependent manner. eLife 11:e73155. 10.7554/eLife.73155.sa2 35968845 PMC9470163

[B23] Ganguly K. Khanna P. Morecraft R. J. Lin D. J. (2022). Modulation of neural co-firing to enhance network transmission and improve motor function after stroke. Neuron 110, 2363–2385. 10.1016/j.neuron.2022.06.024 35926452 PMC9366919

[B24] Goodman G. W. Do T. H. Tan C. Ritzel R. M. (2023). Drivers of chronic pathology following ischemic stroke: a descriptive review. Cell. Mol. Neurobiol. 44:7. 10.1007/s10571-023-01437-2 38112809 PMC11391890

[B25] Hatsopoulos N. G. Suminski A. J. (2011). Sensing with the motor cortex. Neuron 72, 477–487. 10.1016/j.neuron.2011.10.020 22078507 PMC3263767

[B26] Herreras O. (2016). Local field potentials: myths and misunderstandings. Front. Neural Circuits 10:101. 10.3389/fncir.2016.00101 28018180 PMC5156830

[B27] Hill D. N. Mehta S. B. Kleinfeld D. (2011). Quality metrics to accompany spike sorting of extracellular signals. J. Neurosci. 31, 8699–8705. 10.1523/JNEUROSCI.0971-11.2011 21677152 PMC3123734

[B28] Hishinuma A. K. Gulati T. Burish M. J. Ganguly K. (2019). Large-scale changes in cortical dynamics triggered by repetitive somatosensory electrical stimulation. J. Neuroeng. Rehabil. 16:59. 10.1186/s12984-019-0520-1 31126339 PMC6534962

[B29] Hong G. Lieber C. M. (2019). Novel electrode technologies for neural recordings. Nat. Rev. Neurosci. 20, 330–345. 10.1038/s41583-019-0140-6 30833706 PMC6531316

[B30] Hsu W. Y. Cheng C. H. Liao K. K. Lee I. H. Lin Y. Y. (2012). Effects of repetitive transcranial magnetic stimulation on motor functions in patients with stroke. Stroke 43, 1849–1857. 10.1161/STROKEAHA.111.649756 22713491

[B31] Jayaraj R. L. Azimullah S. Beiram R. Jalal F. Y. Rosenberg G. A. (2019). Neuroinflammation: friend and foe for ischemic stroke. J. Neuroinflammation 16:142. 10.1186/s12974-019-1516-2 31291966 PMC6617684

[B32] Jickling G. C. Liu D. Stamova B. Ander B. P. Zhan X. Lu A.. (2014). Hemorrhagic transformation after ischemic stroke in animals and humans. J. Cerebral Blood Flow Metab. 34, 185–199. 10.1038/jcbfm.2013.203 24281743 PMC3915212

[B33] Kilic U. (2025). Figure 1: Experimental Paradigm. Created in BioRender. Available online at: https://BioRender.com/u19amp5

[B34] Kim Y. K. Yang E. J. Cho K. Lim J. Y. Paik N. J. (2014). Functional recovery after ischemic stroke is associated with reduced GABAergic inhibition in the cerebral cortex: a GABA PET study. Neurorehabil. Neural Repair 28, 576–83. 10.1177/1545968313520411 24463186

[B35] Kimberley T. J. Pierce D. Prudente C. N. Francisco G. E. Yozbatiran N. Smith P.. (2018). Vagus nerve stimulation paired with upper limb rehabilitation after chronic stroke. Stroke 49, 2789–2792. 10.1161/STROKEAHA.118.022279 30355189

[B36] Kunori N. Takashima I. (2016). High-order motor cortex in rats receives somatosensory inputs from the primary motor cortex *via* cortico-cortical pathways. Eur. J. Neurosci. 44, 2925–2934. 10.1111/ejn.13427 27717064

[B37] Leonardi G. Ciurleo R. Cucinotta F. Fonti B. Borzelli D. Costa L.. (2022). The role of brain oscillations in post-stroke motor recovery: an overview. Front. Syst. Neurosci. 16:947421. 10.3389/fnsys.2022.947421 35965998 PMC9373799

[B38] Levy R. M. Harvey R. L. Kissela B. M. Winstein C. J. Lutsep H. L. Parrish T. B.. (2016). Epidural electrical stimulation for stroke rehabilitation: results of the prospective, multicenter, randomized, single-blinded everest trial. Neurorehabil. Neural Repair 30, 107–119. 10.1177/1545968315575613 25748452

[B39] Lindén H. Tetzlaff T. Potjans T. C. Pettersen K. H. Grün S. Diesmann M.. (2011). Modeling the spatial reach of the LFP. Neuron 72, 859–872. 10.1016/j.neuron.2011.11.006 22153380

[B40] Loos C. M. Staals J. Wardlaw J. M. van Oostenbrugge R. J. (2012). Cavitation of deep lacunar infarcts in patients with first-ever lacunar stroke. Stroke 43, 2245–2247. 10.1161/STROKEAHA.112.660076 22693127

[B41] Luhmann H. J. Mudrick-Donnon L. A. Mittmann T. Heinemann U. (1995). Ischaemia-induced long-term hyperexcitability in rat neocortex. Eur. J. Neurosci. 7, 180–191. 10.1111/j.1460-9568.1995.tb01054.x 7538854

[B42] Mahlknecht P. Foltynie T. Limousin P. Poewe W. (2022). How does deep brain stimulation change the course of Parkinson's disease? Movement Disord. 37, 1581–1592. 10.1002/mds.29052 35560443 PMC9545904

[B43] Miller E. L. Murray L. Richards L. Zorowitz R. D. Bakas T. Clark P.. (2010). Comprehensive overview of nursing and interdisciplinary rehabilitation care of the stroke patient. Stroke 41, 2402–2448. 10.1161/STR.0b013e3181e7512b 20813995

[B44] Moreau F. Patel S. Lauzon M. L. McCreary C. R. Goyal M. Frayne R.. (2012). Cavitation after acute symptomatic lacunar stroke depends on time, location, and MRI sequence. Stroke 43, 1837–1842. 10.1161/STROKEAHA.111.647859 22733793

[B45] Nayak C. Anilkumar A. (2023). EEG Normal Waveforms. Treasure Island (FL): StartPearls Pubishing.

[B46] Neumann-Haefelin T. Hagemann G. Witte O. W. (1995). Cellular correlates of neuronal hyperexcitability in the vicinity of photochemically induced cortical infarcts in rats *in vitro*. Neurosci. Lett. 193, 101–104. 10.1016/0304-3940(95)11677-O 7478151

[B47] Nowak D. A. Grefkes C. Dafotakis M. Eickhoff S. Küst J. Karbe H.. (2008). Effects of low-frequency repetitive transcranial magnetic stimulation of the contralesional primary motor cortex on movement kinematics and neural activity in subcortical stroke. Arch. Neurol. 65, 741–747. 10.1001/archneur.65.6.741 18541794

[B48] Ordek G. Groth J. D. Sahin M. (2013). Differential effects of ketamine/xylazine anesthesia on the cerebral and cerebellar cortical activities in the rat. J. Neurophysiol. 109, 1435–1443. 10.1152/jn.00455.2012 23236007

[B49] Povysheva N. Nigam A. Brisbin A. K. Johnson J. W. Barrionuevo G. (2019). Oxygen-glucose deprivation differentially affects neocortical pyramidal neurons and parvalbumin-positive interneurons. Neuroscience 412, 72–82. 10.1016/j.neuroscience.2019.05.042 31152933 PMC6818263

[B50] Qü M. Buchkremer-Ratzmann I. Schiene K. Schroeter M. Witte O. W. Zilles K. (1998). Bihemispheric reduction of GABAA receptor binding following focal cortical photothrombotic lesions in the rat brain. Brain Res. 813, 374–380. 10.1016/S0006-8993(98)01063-4 9838197

[B51] Quiquempoix M. Fayad S. L. Boutourlinsky K. Leresche N. Lambert R. C. Bessaih T. (2018). Layer 2/3 pyramidal neurons control the gain of cortical output. Cell Rep. 24, 2799–2807.e4. 10.1016/j.celrep.2018.08.038 30208307

[B52] Rabiller G. He J. W. Nishijima Y. Wong A. Liu J. (2015). Perturbation of brain oscillations after Ischemic stroke: a potential biomarker for post-stroke function and therapy. Int. J. Mol. Sci. 16, 25605–25640. 10.3390/ijms161025605 26516838 PMC4632818

[B53] Ramanathan D. S. Guo L. Gulati T. Davidson G. Hishinuma A. K. Won S. J.. (2018). Low-frequency cortical activity is a neuromodulatory target that tracks recovery after stroke. Nat. Med. 24, 1257–1267. 10.1038/s41591-018-0058-y 29915259 PMC6093781

[B54] Rostami V. Rost T. Schmitt F. J. van Albada S. J. Riehle A. Nawrot M. P. (2024). Spiking attractor model of motor cortex explains modulation of neural and behavioral variability by prior target information. Nat Commun. 15, 6304. 10.1038/s41467-024-49889-4 39060243 PMC11282312

[B55] Roy N. C. Bessaih T. Contreras D. (2011). Comprehensive mapping of whisker-evoked responses reveals broad, sharply tuned thalamocortical input to layer 4 of barrel cortex. J. Neurophysiol. 105, 2421–2437. 10.1152/jn.00939.2010 21325677 PMC3094161

[B56] Rule M. E. Vargas-Irwin C. Donoghue J. P. Truccolo W. (2018). Phase reorganization leads to transient β-LFP spatial wave patterns in motor cortex during steady-state movement preparation. J. Neurophysiol. 119, 2212–2228. 10.1152/jn.00525.2017 29442553 PMC6032117

[B57] Salanova V. Witt T. Worth R. Henry T. R. Gross R. E. Nazzaro J. M.. (2015). Long-term efficacy and safety of thalamic stimulation for drug-resistant partial epilepsy. Neurology 84, 1017–1025. 10.1212/WNL.0000000000001334 25663221 PMC4352097

[B58] Schroeder K. E. Irwin Z. T. Gaidica M. Nicole Bentley J. Patil P. G. Mashour G. A.. (2016). Disruption of corticocortical information transfer during ketamine anesthesia in the primate brain. Neuroimage 134, 459–465. 10.1016/j.neuroimage.2016.04.039 27095309 PMC4912854

[B59] Ting W. K. Fadul F. A. Fecteau S. Ethier C. (2021). Neurostimulation for Stroke Rehabilitation. Front. Neurosci. 15, 649459. 10.3389/fnins.2021.649459 34054410 PMC8160247

[B60] Tsao C. W. Aday A. W. Almarzooq Z. I. Anderson C. A. M. Arora P. Avery C. L.. (2023). Heart Disease and Stroke Statistics-−2023 update: a report from the American Heart Association. Circulation 147, e93–e621. 10.1161/CIR.0000000000001123 36695182 PMC12135016

[B61] Vos T. Lim S. S. Abbafati C.. (2020). Global burden of 369 diseases and injuries in 204 countries and territories, 1990–2019: a systematic analysis for the Global Burden of Disease Study 2019. Lancet 396, 1204–1222. 10.1016/S0140-6736(20)30925-9 33069326 PMC7567026

[B62] Wang J. H. (2003). Short-term cerebral ischemia causes the dysfunction of interneurons and more excitation of pyramidal neurons in rats. Brain Res. Bull. 60, 53–58. 10.1016/S0361-9230(03)00026-1 12725892

[B63] Whishaw I. Q. Dringenberg H. C. Pellis S. M. (1992). Spontaneous forelimb grasping in free feeding by rats: motor cortex aids limb and digit positioning. Behav. Brain Res. 48, 113–125. 10.1016/S0166-4328(05)80147-0 1616602

[B64] Yger P. Spampinato G. L. Esposito E. Lefebvre B. Deny S. Gardella C.. (2018). A spike sorting toolbox for up to thousands of electrodes validated with ground truth recordings *in vitro* and *in vivo*. eLife 7:e34518. 10.7554/eLife.34518.015 29557782 PMC5897014

[B65] Young N. A. Vuong J. Flynn C. Teskey G. C. (2011). Optimal parameters for microstimulation derived forelimb movement thresholds and motor maps in rats and mice. J. Neurosci. Methods 196, 60–69. 10.1016/j.jneumeth.2010.12.028 21219927

[B66] Yu Z. Guindani M. Grieco S. F. Chen L. Holmes T. C. Xu X. (2022). Beyond t test and ANOVA: applications of mixed-effects models for more rigorous statistical analysis in neuroscience research. Neuron 110, 21–35. 10.1016/j.neuron.2021.10.030 34784504 PMC8763600

[B67] Zhang H. Schools G. P. Lei T. Wang W. Kimelberg H. K. Zhou M. (2008). Resveratrol attenuates early pyramidal neuron excitability impairment and death in acute rat hippocampal slices caused by oxygen-glucose deprivation. Exp. Neurol. 212, 44–52. 10.1016/j.expneurol.2008.03.006 18495119 PMC2490603

[B68] Zhao S. Tang X. Tian W.. (2023a). Tracking neural activity from the same cells during the entire adult life of mice. Nat. Neurosci. 26, 696–710. 10.1038/s41593-023-01267-x 36804648

[B69] Zhao Z. Zhu H. Li X.. (2023b). Ultraflexible electrode arrays for months-long high-density electrophysiological mapping of thousands of neurons in rodents. Nat. Biomed. Eng. 7, 520–532. 10.1038/s41551-022-00941-y 36192597 PMC10067539

