# Supplementary material for: Dynamic electrophysiological changes in abnormal brain cavities post-ischemic stroke
Source: Front Neurosci. 2025 May 16;19:1565255. doi: 10.3389/fnins.2025.1565255 (PMC12122521; doi:10.3389/fnins.2025.1565255)
Supplement: Supplementary file 1 [file Data_Sheet_1.docx]

# Supplementary Material

Functional mapping of the motor cortex

We mapped the motor cortex in a separate experiment, using intracortical microstimulation (ICMS) to localize forelimb-associated regions and identify the optimal site for ABC induction. To do this we defined a fixed grid of 88 interconnected sites (each 500 μm²) over the cortical surface. In Figure S1 the coordinate 0,0 refers to bregma (red dot – Figure S1). At each site, a tungsten microTargeting™ electrode (FHC, Inc., USA; 250 μm diameter) was inserted to a depth of 1800 μm to target layer 5. We then applied electrical stimulation which consisted of trains of 13 biphasic (cathodic-first) pulses delivered at 300 Hz, with a pulse width of 100 μs and amplitude of 500 μA following the protocol of (Young et al., 2011). An observer blinded to the stimulation condition recorded visible muscle twitches in the contralateral forelimb. These responses across all rats were used to construct a cortical functional map of the rat forelimb (see Supplementary Figure 1).

**
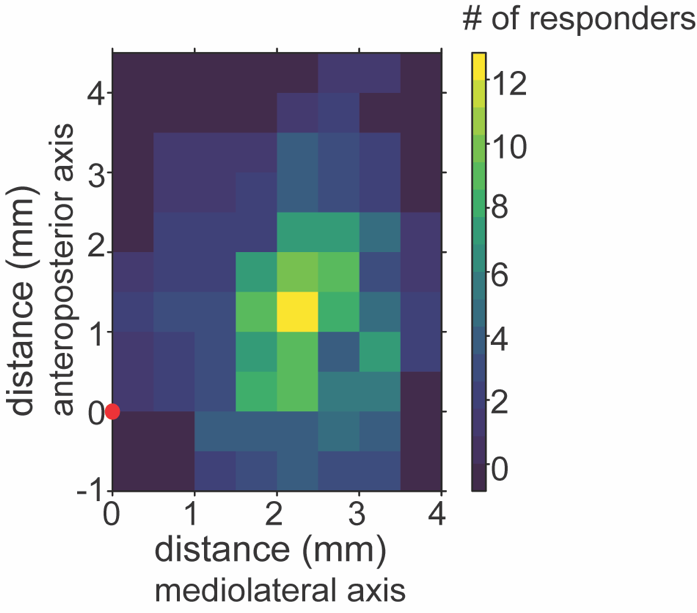
**

Figure S1. **Motor cortex map generated using ICMS.** Each site (a block in the figure) within the 5.5 mm (y-axis corresponding to the anterioposterior dimension) by 4 mm (x-axis corresponding to the mediolateral dimension) surveyed area was stimulated to evoke forelimb movements. Color intensity indicates the number of positive responders. Positive response was defined as visible muscle twitches in the contralateral forelimb upon stimulation. Coordinates are shown relative to bregma and coordinate 0,0 corresponds to Bregma (red dot).


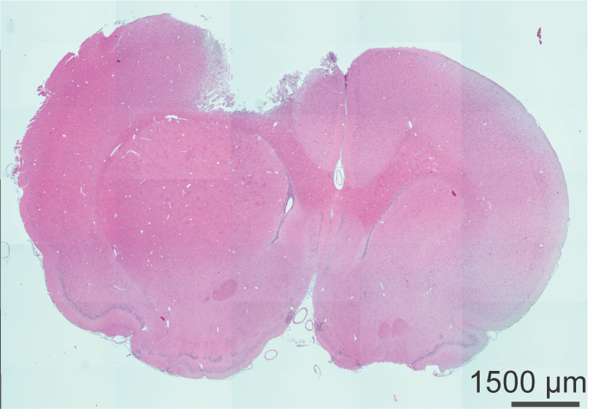


Figure S2. **Individual example of H&E-stained coronal section showing the lesion site within the motor cortex.**
